# The Combination of Whole-Brain Features and Local-Lesion Features in DSC-PWI May Improve Ischemic Stroke Outcome Prediction

**DOI:** 10.3390/life12111847

**Published:** 2022-11-11

**Authors:** Yingwei Guo, Yingjian Yang, Mingming Wang, Yu Luo, Jia Guo, Fengqiu Cao, Jiaxi Lu, Xueqiang Zeng, Xiaoqiang Miao, Asim Zaman, Yan Kang

**Affiliations:** 1 College of Medicine and Biological Information Engineering, Northeastern University, Shenyang 110169, China; 2 College of Health Science and Environmental Engineering, Shenzhen Technology University, Shenzhen 518118, China; 3 Department of Radiology, Shanghai Fourth People’s Hospital Affiliated to Tongji University School of Medicine, Shanghai 200434, China; 4 Department of Psychiatry, Columbia University, New York, NY 10027, USA; 5 School of Applied Technology, Shenzhen University, Shenzhen 518060, China; 6 Engineering Research Centre of Medical Imaging and Intelligent Analysis, Ministry of Education, Shenyang 110169, China

**Keywords:** DSC-PWI, dynamic radiomics features, whole brain, local lesions, lasso, outcome prediction

## Abstract

Accurate and reliable outcome predictions can help evaluate the functional recovery of ischemic stroke patients and assist in making treatment plans. Given that recovery factors may be hidden in the whole-brain features, this study aims to validate the role of dynamic radiomics features (DRFs) in the whole brain, DRFs in local ischemic lesions, and their combination in predicting functional outcomes of ischemic stroke patients. First, the DRFs in the whole brain and the DRFs in local lesions of dynamic susceptibility contrast-enhanced perfusion-weighted imaging (DSC-PWI) images are calculated. Second, the least absolute shrinkage and selection operator (Lasso) is used to generate four groups of DRFs, including the outstanding DRFs in the whole brain (Lasso (WB)), the outstanding DRFs in local lesions (Lasso (LL)), the combination of them (combined DRFs), and the outstanding DRFs in the combined DRFs (Lasso (combined)). Then, the performance of the four groups of DRFs is evaluated to predict the functional recovery in three months. As a result, Lasso (combined) in the four groups achieves the best AUC score of 0.971, which improves the score by 8.9% compared with Lasso (WB), and by 3.5% compared with Lasso (WB) and combined DRFs. In conclusion, the outstanding combined DRFs generated from the outstanding DRFs in the whole brain and local lesions can predict functional outcomes in ischemic stroke patients better than the single DRFs in the whole brain or local lesions.

## 1. Introduction

Stroke has become the second leading cause of death and the third leading cause of disability globally [1]. Nearly 40% of those surviving stroke have a moderate functional impairment, and 15–30% sustain severe permanent disability [2], resulting in impaired quality of life. Thrombolytic therapy is the primary clinical treatment method for ischemic stroke [3,4]. The effect of thrombolytic therapy is mainly determined by the patient’s characteristics and individual treatment plans [5]. If the neurological functional recovery after treatment can be predicted, it will be helpful to make the appropriate treatment decision. Therefore, it is desirable to accurately predict the prognosis and rehabilitation of patients to assist physicians in making suitable treatment programs for patients and reducing their adverse prognosis. 

The modified Rankin Score (mRS), ordered from 0 (no symptoms at all) to 6 (death), is the most widely used functional outcome measure for stroke. An mRS from 0 to 1 is defined as a good outcome, while scores 2 to 6 are defined as a poor outcome in the clinic [6]. Previous studies have identified clinical text information (CTI) from patients and medical images associated with stroke outcomes. For example, Refs. [7,8,9,10] proved that basic patient information, physical symptoms, clinical scores, and features in damaged tissue could predict stroke outcome, and could especially predict the modified Rankin Score (mRS) in three months. Ref. [11] provided an additional feature selection mechanism to select essential features from the CTI for improving outcome prediction. Ref. [12] developed a nomogram model with 12 independent predictors of patients to identify the mortality in intensive care unit patients with stroke. Although the recovery status of stroke patients can be preliminarily predicted through the CTI, it is difficult to improve the prediction performance due to the limitation of missing the information reflecting patients’ cerebral blood flow (CBF) status.

Recently, the significant value of medical images in predicting stroke outcomes has been revealed. For example, multiple-category prediction was achieved using the hemodynamic parameters computed from perfusion images provided by ISLES 2016 and 2017 [13,14,15]. In addition, some scholars used collateral vessels on computed tomography angiography (CTA) to predict outcomes [16]. The added value of computed tomography perfusion (CTP) and CTA parameters in outcome prediction of acute stroke patients were proved in Ref. [17]. However, although clinical control variables computed from medical images have been widely used in the functional recovery prediction of stroke patients, the stable value of medical images in predicting outcomes is not clear-cut [18]. Therefore, it is necessary to develop new intelligent methods to improve the value of medical images in stroke outcome prediction.

Radiomics refers to the automated extraction of quantifiable data from radiological images, and the extracted features can be used in predicting clinical outcomes [19,20,21,22]. Refs. [23,24] concluded that the radiomics signatures obtained by feature selection have better performance than those without feature selection. Moreover, after exploring the application of radiomics features of three-dimensional (3D) images, the radiomics features of four-dimensional (4D) perfusion images containing blood flow information began to be recognized and applied to outcome prediction. Some researchers combined CTI and radiomics features to improve the outcome prediction. Ref. [25] extracted radiomics features from 3D images in time series of dynamic susceptibility contrast-enhanced perfusion-weighted imaging (DSC-PWI) to generate dynamic radiomics features (DRFs), and the performance of DRFs, CTI, generated survival features, and the combination of them were compared and evaluated. The results showed that the CTI performed better than DRFs of ischemic lesions, and the additional survival features can improve the predictive ability. Based on the above, it can be concluded that the current research in prognosis prediction mainly focuses on features of local lesion regions in medical images. However, it should not be ignored that the appearance of local ischemic lesions will inevitably lead to changes in the cerebral blood flow parameters in the whole brain [26,27]. Both local lesions and the whole brain should imply neurological impairment and recovery ability [28]. Therefore, the comparative and exploration of the influence of whole-brain features and local-lesion features on the outcome may provide more possibilities for the clinical treatment and prognosis prediction of ischemic stroke patients.

This study investigated and validated the role of dynamic radiomics features (DRFs) in the local lesions and the whole brain of DSC-PWI images for predicting ischemic stroke outcomes (90-day mRS). The outstanding DRFs in the whole-brain and local-lesion regions were extracted and combined, and the combined DRFs successfully improved the outcome prediction compared to the individual DRFs. This study is expected to be a potential tool in the clinic. 

## 2. Materials and Methods

A detailed introduction of the materials and methods is listed below. The provided methods mainly consist of five steps: (1) preprocessing datasets, (2) segmentation of the whole brain and ischemic lesion, (3) calculating DRFs and *t*-test analysis, (4) feature selection and combination, and (5) ischemic stroke outcome prediction.

### 2.1. Materials

The datasets were collected from 2013 to 2016 by the Neurology Department of Shanghai Fourth People’s Hospital Affiliated with Tongji University School of Medicine, China. The datasets reviewed 78 DSC-PWI images from 56 consecutive patients with ischemic stroke. Their neurological function recovery was redefined depending on the 90-day mRS scored by an experienced physician. In detail, the 90-day mRS was divided into good outcomes and poor outcomes. A score greater than 2 was defined as a poor outcome, while the opposite situation was defined as a good outcome. Then, 42 cases with good outcomes and 36 with poor outcomes were obtained. Moreover, all DSC-PWI images were scanned within 24 h of symptom onset by the 1.5T MR scanner (Siemens, Munich, Germany). Table 1 shows the details of the DSC-PWI datasets.

### 2.2. Methods

#### 2.2.1. Preprocessing Datasets

**Figure 1:**
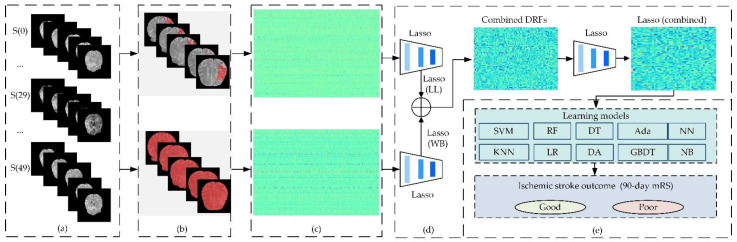
Flowchart of the provided method in this study: (**a**) preprocessed DSC-PWI images; (**b**) segmentation of the whole brain and ischemic lesion; (**c**) computed dynamic radiomics features from the whole brain and ischemic lesion; (**d**) feature selection and combination; (**e**) ischemic stroke outcome prediction.

Figure 1 shows the flowchart of our provided methods. The first work in this study was to reduce noise and position deviation in DSC-PWI datasets. With the multiplicative intrinsic component optimization algorithm introduced in Refs. [29,30], the image series in DSC-PWI data were registered, and the potential patient motion was reduced. Next, this study used neuroimaging software package FSL [31] to segment the skull from the average 3D DSC-PWI image, then obtain the brain tissue region of DSC-PWI images. Since the time period of the contrast agent passed through is in the range of about 17 to 22 and the end of the reaction is located at a time value greater than 30 [25], the average 3D image was computed from the first 15th and the last 15th 3D images to reduce the influence of the contrast agent. The computation of the average 3D image is expressed as Equation (1).

(1) $$\mathrm{avg}\_\mathrm{pwi}=\frac{\overset{n}{\underset{i=1}{\sum }}S(i)+\overset{N}{\underset{i=N-n}{\sum }}S(i)}{2n}$$

where *avg_pwi* is the average 3D image, *N* is the total measurements of DSC-PWI (*N* = 50 in this study), *n* = 15, and *S*(*i*) is the *i*th 3D image in the DSC-PWI sequence.

Moreover, we used the data smoothing filter with a 1 × 3 filtering kernel introduced in Ref. [25] to smooth DSC-PWI images and decrease their noise. Then, the time–intensity curve of each voxel in the DSC-PWI image was smoothed. This way, the DSC-PWI images in the datasets included the smoothed 3D images with 50 measurements.

#### 2.2.2. Segmentation of the Whole Brain and Ischemic Lesion

**Figure 2:**
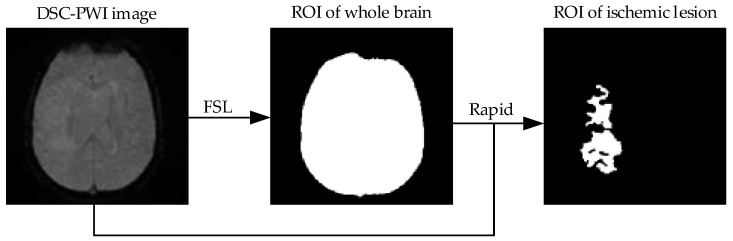
Flowchart of making ROIs of whole brain and ischemic lesion.

The primary task for comparative analysis of features in the whole brain and local lesions is to detect both regions. The regions of interest (ROIs) of the whole brain in the DSC-PWI images were obtained based on the result of skull segmentation in Section 2.2.1. Depending on the software FSL, the brain tissue mask and skull mask can be obtained, and then the brain tissue mask can be regarded as the ROI of the whole brain in the DSC-PWI image. For the detection of ischemic stroke lesions, the Rapid Processing of Perfusion and Diffusion (RAPID) software (iSchemaView, Menlo Park, CA, USA) [32] was applied to make the ROI of the ischemic stroke lesions in the whole brain, and the set condition was Tmax > 6 s. Then, the ROIs of local ischemic lesions and the whole brain can be obtained for each DSC-PWI image (seen in Figure 2).

#### 2.2.3. Calculating DRFs and Selecting Significant Radiomics Features by *t*-Test Analysis

**Figure 3:**
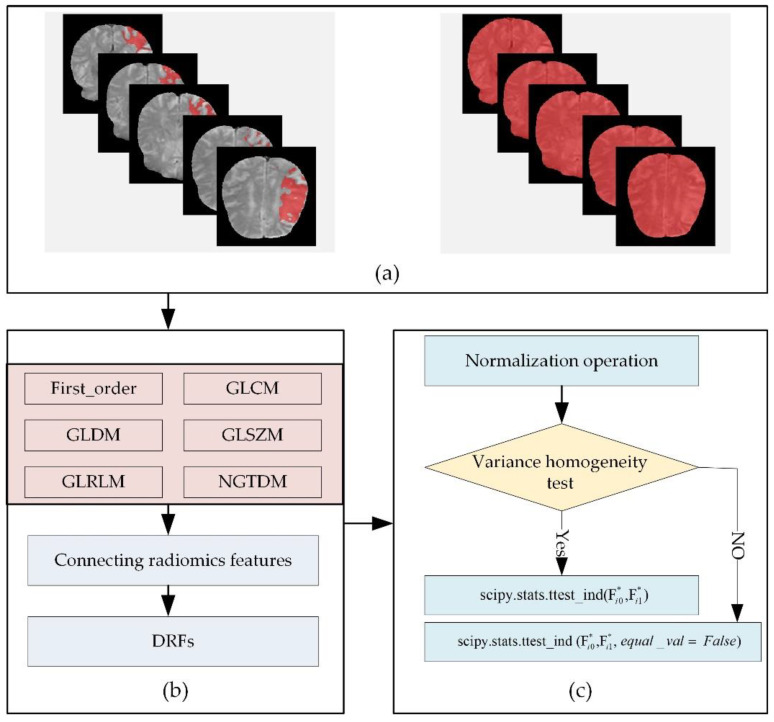
Flowchart of calculating radiomics features and selecting significant radiomics features: (**a**) DSC-PWI images and ROIs of the whole brain and local lesions; (**b**) calculating DRFs for ROIs in DSC-PWI image, and (**c**) extracting significant DRFs by *t*-test analysis.

Due to the 4D characteristics of the DSC-PWI images, this study split them into N 3D images to process separately. In detail, this study used radiomics technology to calculate the radiomics features of the whole brain and local lesions in each 3D image (Figure 3a). The radiomics features were combined according to the time order to generate the dynamic radiomics features (DRFs). That means the radiomics features of the whole brain and ischemic tissue in each 3D image could be computed separately. Then, the radiomics features list can be generated by combining the radiomics features of all the 3D images in the time order. The combined radiomics features were defined as DRFs of the DSC-PWI images.

Among the DRFs, there were six feature groups: First-Order Statistics (First_order), Gray-Level Dependence Matrix (GLDM), Gray-Level Co-Occurrence Matrix (GLCM), Gray-Level Run-Length Matrix (GLRLM), Gray-Level Size-Zone Matrix (GLSZM), and Gand Neighboring Gray-Tone Difference Matrix (NGTDM) (Figure 3b). In this study, radiomics features were automatically calculated by using the PyRadiomics package implemented in Python [33,34]. The 3D image in the DSC-PWI was defined as S(n), n ∈ [0, 49], and the calculated DRFs were defined by combining their radiomics feature names and the n-values of the 3D images. For example, “wavelet-HHL_gldm_SmallDependenceLowGrayLevelEmphasis_1” represents the radiomics feature “wavelet-HHL_gldm_SmallDependenceLowGrayLevelEmphasi” of 3D image S(1), which is the second 3D image (the first one is S(0)) in DSC-PWI data, and this feature belongs to the GLDM group and filtered by wavelet.

Before feature selection, a feature normalization is necessary to remove the influence of differences between samples and features. This study used each feature’s mean and standard deviation to normalize the feature vector. The transformation is given in Equation (2).

(2) $$F_{i}^{*}=\left(F_{i}-{\bar{F}}_{i}\right)/\left(F_{\mathrm{imax}\;}-F_{\mathrm{imin}\;}\right)$$

where $F_{i}^{*}$ is the updated normalized result of *F_i_*; ${\bar{F}}_{i}$, *F_imax_*, and *F_imin_* represent the mean, maximum, and minimum of *F_i_*; and *i* is the order of DRF.

Then, this study used the *t*-test algorithm to extract the significant DRF (Figure 3c). First, this study detected the homogeneity of variance of each DRF. When obtaining a positive result that the feature had homogeneity of variance, the *t*-test was performed directly. However, if obtaining a negative result, this study set the parameter equal_val as false when performing *t*-test analysis. Last, the significant DRFs (*p* < 0.05) remained in the *t*-test analysis. Therefore, two groups of significant DRF from the whole brain and local lesions were obtained, and they were defined as T-test (WB) and T-test (LL) in this study. Moreover, the Levene test was used to realize the homogeneity of the variance test.

#### 2.2.4. Feature Selection and Combination

Feature selection plays a crucial role in data mining and improving algorithms performance, which is necessary work in feature processing. The Lasso algorithm has been recognized as one of the most effective selection methods for selecting relevant features with target variables [25,35,36]. Therefore, this study used Lasso to select outstanding radiomics features for 90-day mRS, and the feature with a non-zero coefficient was selected as one of the outstanding DRFs. The Lasso was implemented by the LassoCV function imported from sklearn.linear_model package in Python 3.6, and the cv was set as 10 in the function. The mathematical principle of Lasso is shown in Equation (3). By the supervised feature selection, outstanding DRFs can be obtained according to the ground truth of 90-day mRS. The outstanding DRFs selected from original radiomics features of the whole brain were defined as Lasso (WB), while those of local lesions were defined as Lasso (LL).

(3) $$Lasso\left(F_{t-\mathrm{test}})=\mathrm{argmin}\{\overset{M}{\underset{i=1}{\sum }}{\left(y_{i}-\beta_{0}-\overset{q}{\underset{j=1}{\sum }}\beta_{j}x_{ij}\right)}^{2}+\lambda \overset{q}{\underset{j=0}{\sum }}|\beta_{j}|\right\}$$

where, Lasso (*F_t-test_*) represents the selected outstanding DRFs for the outcome prediction from the significant DRF *F_t-test_*; *x_ij_* is the independent DRF in *F_t-test_*; *y_i_* is the ground truth of the *i*th case; *λ* is the penalty parameter greater than zero; *β_j_* is the regression coefficient; *M* represents the number of samples for the selection operation; *q* represents the selected DRFs by the Lasso algorithm; *i* ∈ [1, *M*]; and *j* ∈ [0, *q*].

Then, this study combined the DRFs from both regions and regarded them as combined DRFs (Lasso (WB) + Lasso (LL), ‘+’ means the concatenation operation of features). Furthermore, this study used the Lasso algorithm to perform feature extraction on the combined DRFs, and the outstanding combined features defined as Lasso(combined) were obtained.

#### 2.2.5. Ischemic Stroke Outcome Prediction

(1) Experiments

To verify the proposed methods, we used the methods to process the DSC-PWI datasets. The outcome prediction performance of outstanding DRFs in the whole brain, outstanding DRFs in the local lesions, combined DRFs, and outstanding combined DRFs can be obtained. Then, their performance can be calculated.

(2) Evaluating the Performance of Outcome Prediction

This study used ten machine learning models to evaluate four groups of DRFs. By training the ten models with the four groups of features (Lasso (WB), Lasso (LL), combined DRFs (Lasso (WB) + Lasso (LL)), and Lasso (combined)), the area under the curves (AUCs) of ten machine learning models can be computed, respectively. According to the AUCs, the ability of four groups of outstanding DRFs to predict stroke outcomes can be examined. For the ten models, this study selected the ten most widely used learning models for the classification task in data mining and intelligent analysis [37,38,39]. In detail, the ten learning models are listed in Table 2 and implemented by Python 3.6, including GaussianNB (NB), logistic regression (LR), decision tree (DT), gradient boosting classifier (GBDT), multilayer perceptual neural network (nn), k-nearest neighbors (KNN), Adaboost classifier (Ada), linear discriminant analysis (DA), random forest (RF), and support vector machine (SVM). 

When performing the tenfold cross-validation, the StratifiedKFold function imported from the sklearn package was used to ensure the same proportion of positive and negative samples in the training and test sets.

## 3. Results

The detailed results of the provided methods are divided into four parts, including calculated DRFs and selected significant DRFs, generated four groups of radiomics features by Lasso, and the outcome prediction performance. A description of the detailed results is provided below.

### 3.1. Computed DRFs and Selected Significant DRFs

#### 3.1.1. Computed DRFs

**Figure 4:**
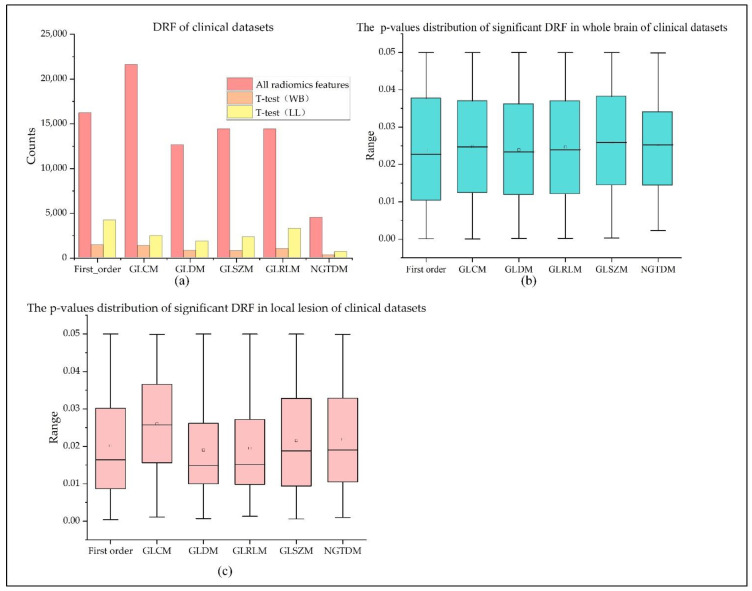
Distribution of DRFs in original radiomics features and significant radiomics features: (**a**) counts of all the DRFs and significant DRFs in the whole brain and local lesions; (**b**,**c**) box plots of *p*-values of significant DRFs of the whole brain and local lesions.

For each 3D image in the 4D DSC-PWI image, 1674 radiomics features belonging to six radiomics feature groups can be calculated. These six groups of radiomics features are 324 first-order features, 432 GLCM features, 288 GLRLM features, 288 GLSZM features, 90 NGTDM features, and 252 GLDM features. Thus, 83,700 DRFs (50 measurements × 1674 features) for the DSC-PWI image can be obtained (see Figure 4a).

#### 3.1.2. Selected Significant DRF of Whole Brain and Local Lesions

By the *t*-test analysis, this study extracted 5564 significant DRFs from the whole brain, and there were 1410 DRFs in First_order with *p*-values of 0.024 ± 0.015, 1332 DRFs in GLCM (0.025 ± 0.015), 782 DRFs in GLDM (0.024 ± 0.014), 1000 DRFs in GLRLM (0.025 ± 0.015), 752 DRFs in GLSZM (0.026 ± 0.014), and 288 DRFs in NGTDM (0.025 ± 0.012). Moreover, this study extracted 14661 significant DRFs from the local lesions, and there were 4177 DRFs in First_order with *p*-values of 0.02 ± 0.014, 2445 DRFs in GLCM (0.026 ± 0.013), 1830 DRFs in GLDM (0.019 ± 0.013), 3248 DRFs in GLRLM (0.02 ± 0.012), 2303 DRFs in GLSZM (0.022 ± 0.014), and 658 DRFs in NGTDM (0.022 ± 0.014). The detailed statistics of each significant DRF were introduced in Table 3 and Table 4 and Figure 4. 

### 3.2. Selected Outstanding DRF and Combined DRF

**Figure 5:**
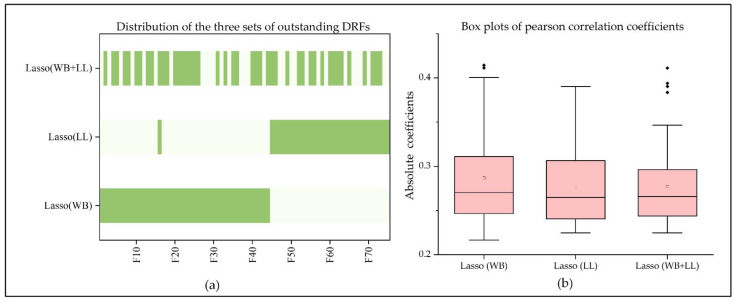
Distribution of outstanding DRFs in the whole brain, local lesions, and both regions: (**a**) outstanding DRFs of the whole brain, local lesions, and both regions, where F1-F70 are the outstanding DRFs series; (**b**) box plots of the absolute pearson correlation coefficients of outstanding DRFs extracted from the whole brain, local lesions, and both regions.

By Lasso analysis, 44 outstanding DRFs were selected from significant DRFs of the whole brain, and there were 9 DRFs in First_order, 15 DRFs in GLCM, 8 DRFs in GLDM, 5 DRFs in GLRLM, 2 DRFs in GLSZM, and 5 in NGTDM. The Pearson coefficients of the selected outstanding DRFs and 90-day mRS ranged from 0.287 ± 0.053. Moreover, 32 outstanding DRFs were selected from significant DRFs of the local lesions, and there were 7 DRFs in First_order, 15 DRFs in GLCM, 9 DRFs in GLSZM, and 1 DRF in NGTDM. The Pearson coefficients of the selected outstanding DRFs and 90-day mRS ranged from 0.278 ± 0.047. Comparing 44 outstanding DRFs of the whole brain and 32 outstanding DRFs of local lesions, only one feature, ‘log-sigma-3-0-mm-3D_glszm_SmallAreaLowGrayLevelEmphasis_13’ was in both sets. Based on the results, this study redefined the selected DRFs by connecting the character ‘F’ and the order of the feature. All selected outstanding DRFs were F1 to F75, in which F16 was the common DRF (see Table 3 and Table 4 and Figure 5a,b). By combining the outstanding DRFs in the whole brain and local lesions, 76 DRFs were obtained, and 45 DRFs among them were selected by Lasso and regarded as Lasso (combine).

### 3.3. Performance of Four Groups of DRFs for Predicting Ischemic Stroke Outcome

This study validated the ability of outstanding DRFs in the whole brain (Lasso(WB)), outstanding DRFs in local lesions (Lasso (LL)), and the combination of them in the outcome prediction of stroke patients. 

Table 5 shows the AUCs of the four groups of DRFs on the ten models. The outstanding DRFs in the local lesions (Lasso (LL)), the outstanding DRFs in the whole-brain (Lasso (WB)), combined DRFs (Lasso (WB) + Lasso (LL)), and outstanding combined DRFs (Lasso (combined)) obtained AUCs of 0.802 ± 0.069, 0.826 ± 0.088, 0.820 ± 0.117, and 0.838 ± 0.142, respectively. For the four groups of DRFs, the best AUC achieved by Lasso (LL) was 0.882, while Lasso (WB) and combined DRFs achieved the same best AUC of 0.936, and Lasso (combined) got the best AUC of 0.971 which was the best score in the four groups. Among the ten models, except for models Ada, GBDT, and DA, Lasso (WB) performed better than Lasso (LL) on the other models. When using Lasso (WB) and Lasso (LL) to obtain the combined DRFs, the AUC was improved on some models, but the best AUC was still 0.936. However, when selecting Lasso (combined) from combined DRFs, the best AUC was raised to 0.971. According to the above result, it can be found that both the information in the whole brain tissue and ischemic tissue can reflect the functional outcome of stroke patients, and in some specific models, whole-brain-features Lasso (WB) containing local lesion information achieved better than local-features Lasso (LL) themselves. Although the direct combining of them failed to improve the prediction performance, it was effectively improved after the lasso algorithm was used to extract Lasso (combined), revealing the linear relationship between Lasso (WB) and Lasso (LL).

In general, SVM, nn, and LR performed better than others of all ten models, and the performance rules of the four groups of DRFs on these three models are Lasso (combined) > combined DRFs ≥ Lasso (WB) > Lasso (LL).

As described above, a previous study [26] used the survival features SurvF to improve the outcome prediction by combining SurvF with the CTI and DRFs extracted from the ischemic lesions. The best performance of the combination of CTI and SurvF (CTI + SurvF), and the combination of the CTI, DRFs in ischemic lesions and SurvF (ALL) are listed in Table 5. The best AUC achieved by Ref. [26] was 0.949, which was lower than the best AUC of Lasso (combined).

## 4. Discussion

Physicians often make treatment decisions for stroke patients based on their judgment about whether the patient will benefit from the designed treatment. Over the years, many challenges and attempts have been made to realize the accurate outcome prediction for ischemic stroke [40,41]. Most previous studies have been based on the clinical information of patients (sex, age, hypertension, diabetes, dizziness, etc.) and imaging information of stroke lesions (volume, image features) to carry out predictive analysis of stroke. However, it has been certified that the CBF parameters are damaged in the regions surrounding or in the stroke lesions [42,43,44]. Recently, Refs. [45,46] have proved that brain changes after stroke are not only characterized by regional changes in the lesion region. Therefore, it is reasonable to believe that the functional recovery of stroke patients is not only related to the condition of the local lesion region but also the information in other regions or the whole brain can affect or characterize the recovery of patients. This study investigated and validated the significance of the DRFs in local lesions and the whole brain for predicting ischemic stroke prognosis. As a result, the outstanding combined DRFs extracted from the whole brain and local lesions achieved an AUC of 0.971. In theory, physicians could eventually use the methods to predict outcomes for a stroke patient by combining imaging variables in the whole-brain and local-lesion regions. Therefore, this study is expected to contribute to clinical practice and provide a clinical tool for physicians.

Medical images, such as CTP and PWI, have been widely used in stroke detection, lesion segmentation, final infarct prediction, and prognosis assessment [27,43,47,48,49], proving that these imaging features are closely related to the current and future rehabilitation of stroke patients. Although the ability of features in stroke lesions to reflect functional recovery has been revealed [23,24,25], few studies are related to the role of whole-brain features in stroke outcome prediction. Under the inspiration of Ref. [25], predicting outcome with the DRFs extracted from ischemic lesion regions, and Ref. [28], using the DRFs in the whole brain to detect stroke and predict outcomes, this study innovatively combined the DRFs of local lesions and the whole brain to explore their contribution to prognosis. As a result, the performance of DRFs in the whole brain (best AUC = 0.936) was better than the DRFs in the local lesions (best AUC = 0.882). It can be concluded that compared with the local-lesion region, the whole-brain features may provide additional information in assessing the recovery of neurological function. Some studies have demonstrated that the abnormalities in brain regions may bring about changes in multiple regions [26,27]. This means that the appearance of local ischemic lesions will inevitably lead to changes in the CBF parameters in the whole brain. Both local lesions and the whole brain contain information reflecting neurological impairment and recovery ability. This study also proved the above viewpoints and concluded that the whole-brain features could better reflect the prognosis. Moreover, based on the distribution of DRFs shown in Figure 5, the outstanding DRFs in the whole brain and local lesions are relatively independent. That means the features in global and local regions that affect the patient’s ability to function are differentiated. Thus, it is necessary to separately analyze the features of both groups in predicting stroke outcomes.

Furthermore, to make further improvements for the outcome prediction, this study attempted to combine the outstanding DRFs selected from the whole brain and local lesions and compare the performance of the combined DRFs and the outstanding DRFs in the combined DRFs (Lasso (combined)). It is gratifying to note that both the combined DRFs and the outstanding combined DRFs can improve the results in some models, and Lasso (combined) achieved the best AUC of 0.971. Thus, the linear relationship of DRFs in both the whole brain and local lesions and the functional recovery of stroke patients can be established. Therefore, the outstanding combined DRFs can be used to predict stroke outcomes effectively. 

There are some limitations of this study. First, the size of the datasets is relatively small, and the ratio of female patients to male patients is disproportionate. As for the fact that there are 78.2% male patients in the datasets, a previous study [50] has proved that sex differences in stroke outcomes are slight. Ref. [51] indicated women fared poorly compared to men, but this negative prognostic sex effect was neutralized by thrombolysis. Therefore, it can be seen that the imbalance of sex has little influence on the outcome prediction. Moreover, depending on the statistics of the datasets, the patients with good and poor outcomes were 42 and 36, respectively. The number of positive and negative samples is similar, reducing the possibility of inadequate training. Under the weak influence of the gender gap and balanced sample, the datasets of this study can be used for classification prediction research, and the results are credible. Furthermore, since this study aims to evaluate the role of the combined DRFs of the whole brain and local lesions in predicting the prognosis of stroke, only imaging features (DRFs) extracted from DSC-PWI images were involved. That means the basic information of patients (age, gender, medical history, etc.) and other clinical text information were not regarded as the evaluation factors but a comparison with the method proposed in this study. As shown in Table 5, the best AUC score in Ref. [24] using the CTI and DRFs in the ischemic lesions was 0.949, while the best AUC in our study was 0.971. Thus, the method of this study is outstanding in terms of results. In addition, ten machine learning models were used to perform the validation. The result is credible and better. According to the results in Table 5, SVM, nn, LR, and NB obtained the same performance pattern, Lasso (combined) > combined DRFs ≥ Lasso(WB) > Lasso(LL), and nn and LR achieved the best AUC score of 0.971. Since the results of multiple models are consistent, it can be proved that the results of this study are not accidental. Furthermore, when evaluating the performance of the four groups of DRFs (Lasso (LL), Lasso (WB), combined DRFs, and Lasso (combined)), the tenfold cross-validation was performed to reduce the influence of sample size. The StratifiedKFold function used in the tenfold cross-validation ensured an equal proportion of positive and negative samples in training and test sets. Thus, the stability of the evaluation results can be improved through balanced sample distribution and cross-validation. Based on the above, the provided method of this study is feasible, the results are referable and credible, and it has potential application value for clinical practice. 

Another limitation is our method’s limited complexity. The role of DRFs extracted from the local ischemic region and the whole brain in predicting stroke outcomes has been proved in Refs. [25,28]. Both of them are preliminary works of this study. In Ref. [25], the CTI, DRFs extracted from the ischemic lesions, and survival features were fused and compared. As it turned out, the performance of the CTI was better than the DRFs. When combining the survival features with the CTI or DRFs, the performance can be improved, and the best score in predicting good and poor outcomes was 0.949. In Ref. [28], the supervised feature selection (Lasso) and unsupervised feature selection methods (five-feature dimension-reduction algorithms) were used to generate different combinations of DRFs, to improve the performance. Although previous studies have proved that the DRFs in local lesions or the DRFs in the whole brain can be used for prognostic prediction, different feature processing methods or novel features are necessary to improve the predictive ability when the two are used alone. In that case, this study intended to evaluate the role of the combined DRFs in the whole brain and local lesions in predicting the prognosis of stroke without using other complex fusion methods. Although the method is not particularly complicated, the results show that the prediction performance is significantly improved by combining lesion and whole-brain features, which was better than the previous study Ref. [25]. Thus, even though this study’s innovation is to combine the DRFs of local lesions and the whole brain, its excellent performance cannot be ignored. Although the results showed that the outstanding combined DRFs selected from whole-brain DRFs and local-lesion DRFs could improve the performance of outcome prediction, further validation should be performed before the methods are used in the clinical setting. We will validate and improve our provided methods with more extensive and diverse datasets in the future. This study is expected to become the guiding tool for physicians in making stroke treatments.

## 5. Conclusions

In conclusion, this study may contribute to guiding physicians to make clinical decisions for ischemic stroke patients. First, the outstanding DRFs in the whole brain and local lesions can predict ischemic stroke outcomes. Second, the performance can be improved when combining the DRFs in the whole-brain and local-lesion regions. Moreover, the outstanding combined DRFs extracted from the combined DRFs can improve the predictive performance further. Because the classifiers used in this study have different attributes, their performance varies. Among the ten learning models, SVM, nn, LR, and NB achieved better performance than others in our study. Thus, model selection is necessary when performing predictions.

## Figures and Tables

**Table 1:** Patient information and DSC-PWI images parameters.

	Item	Parameter
Patient information	Patients	78
	Male	61 (78.21%)
	Age	71.68 ± 10.66
	90-day mRS	2.60 ± 2.34
DSC-PWI images	Matrix	256
	Slices	20
	Number of measurements	50
	Thickness	6.5 mm

**Table 2:** Definition and implementation of the ten models in this study.

Classifier	Implementation in Python 3.6
NB	sklearn.naive_bayes. GaussianNB()
LR	sklearn.linear_model.logisticRegressionCV(max_iter = 100,000, solver = “liblinear”)
DT	sklearn.tree. DecisionTreeClassifier()
GBDT	sklearn.ensemble.GradientBoostingClassifier()
nn	sklearn.neural_network. MLPClassifier (hidden_layer_sizes = (400, 100), alpha = 0.01, max_iter = 10,000)
KNN	sklearn.neighbors. sklearn.neighbors()
Ada	sklearn.ensemble.AdaBoostClassifier()
DA	sklearn.discriminant_analysis()
RF	sklearn.ensemble.RandomForestClassifier(n_estimators = 200)
SVM	sklearn.svm.SVC(kernel = ‘rbf’,probability = True)

**Table 3:** Descriptions of the obtained DRFs in each feature processing method.

Item	First_Order	GLCM	GLDM	GLSZM	GLRLM	NGTDM	Sum
All DRF	16,200	21,600	12,600	14,400	14,400	4500	83,700
T-test (WB)	1410	1332	782	752	1000	288	5564
T-test (LL)	4177	2445	1830	2303	3248	658	14,661
Lasso (WB)	9	15	8	2	5	5	44
Lasso (LL)	7	15	0	9	0	1	32
Combined DRFs	10	16	5	8	2	4	45

**Table 4:** Statistics of the significant DRFs in the whole brain and local lesions.

DRFs	Group	Mean	Std	Sum	Minimum	Medium	Maximum
T-test (WB)	First_order	0.024	0.015	33.57	<0.0001	0.023	0.050
	GLCM	0.025	0.015	32.986	<0.0001	0.025	0.050
	GLDM	0.024	0.014	18.69	<0.0001	0.023	0.050
	GLRLM	0.025	0.015	18.538	<0.0001	0.024	0.050
	GLSZM	0.026	0.014	25.912	<0.0001	0.026	0.050
	NGTDM	0.025	0.012	7.322	0.002	0.025	0.050
T-test (LL)	First_order	0.020	0.014	84.315	0.000	0.016	0.050
	GLCM	0.026	0.013	63.610	0.001	0.026	0.050
	GLDM	0.019	0.013	34.826	0.001	0.015	0.050
	GLRLM	0.020	0.012	44.914	0.001	0.015	0.050
	GLSZM	0.022	0.014	69.973	0.001	0.019	0.050
	NGTDM	0.022	0.014	14.393	0.001	0.019	0.050

**Table 5:** Performance of four groups of outstanding DRFs.

Models	Lasso (WB)	Lasso (LL)	Combined DRFs	Lasso (Combined)	CTI + survF in Ref. [26]
SVM	0.907	0.857	0.923	0.948	0.897
nn	0.923	0.844	0.923	0.971	0.882
RF	0.802	0.784	0.801	0.801	0.949
DT	0.729	0.659	0.676	0.556	0.890
KNN	0.869	0.844	0.911	0.9	0.851
Ada	0.698	0.753	0.738	0.718	0.918
LR	0.936	0.882	0.923	0.971	0.892
NB	0.886	0.857	0.936	0.942	0.821
GBDT	0.74	0.744	0.722	0.691	0.908
DA	0.772	0.798	0.643	0.879	0.790
Mean ± Std	0.826 ± 0.088	0.802 ± 0.069	0.820 ± 0.117	0.838 ± 0.142	0.880 ± 0.047

## Data Availability

Data supporting this study’s findings are available from the corresponding author upon reasonable request.
